# Development of a vaccine delivery system using hepatitis B core antigen based VLPs to deliver mycobacterial antigens

**DOI:** 10.1186/1471-2334-12-S1-P9

**Published:** 2012-05-04

**Authors:** Dhananjayan Dhanasooraj, R Ajay Kumar, Sathish Mundayoor

**Affiliations:** 1 Mycobacterium Research Group, Rajiv Gandhi Centre for Biotechnology, Thiruvananthapuram, Kerala, India

## Background

Growing prevalence of TB and the emergence of XDR-TB have stimulated substantial efforts to develop better vaccines for TB. Recent researches have shown that some of the antigenic proteins and fusion of different proteins produced by *Mycobacterium tuberculosis* can give protection in animal models when administrated with specific adjuvants. In the present study, we explored the use HBcag-VLPs for delivery of tuberculosis antigens.

## Methods

HBcVLPs bearing ESAT-6 and CFP-10 were constructed using PCR and recombinant DNA methods. Proteins were expressed in *E. coli* and purified. VLPs formation was confirmed with TEM. BALB/c mice were immunized with VLPs and controls without any adjuvants. Sera were analysed for antibody responses (ELISA). Splenocytes were cultured and restimulated with purified antigens and CF (culture filtrate) of M.tb. The cell proliferation was measured using cell proliferation assay kit and the culture supernatants were analysed for IL-2, IFN-γ and TNF.

## Results

The recombinant VLP induces preferentially a Th1-type immune response against mycobacterial antigen even though Th2 has been reported as the predominant response in BALB/c mice. IFN-γ, IL-2, TNF and proliferation were significantly higher in mice immunised with HBcVLPs-*M. tuberculosis* antigen. Restimulation with mycobacterial CF also produced the same effect.

## Conclusion

The humoral and cellular responses suggest that the VLP containing fusion constructs generated immune response in a Th1 dependent manner. By virtue of its self-adjuvant nature, HBc VLPs are a better vaccine delivery system for use with newer antigens identified in the course of recent developments in subunit protein vaccine research in tuberculosis.

